# Multicentre experience with the variable-loop circular catheter pulsed-field ablation system for atrial fibrillation ablation

**DOI:** 10.1093/europace/euag265

**Published:** 2026-09-16

**Authors:** Antonio Dello Russo, Yari Valeri, Giuseppe Ciconte, Marco Schiavone, Paolo Compagnucci, Sara Poggi, Raffaele Salerno, Antonio Di Monaco, Lorenzo Gigli, Daniela Aschieri, Stefania Riva, Matteo Anselmino, Livio Bertagnolli, Luca Rossi, Patrizio Mazzone, Giuseppe Stabile, Michela Casella, Massimo Grimaldi, Claudio Tondo, Carlo Pappone

**Affiliations:** Department of Biomedical Sciences and Public Health, Marche Polytechnic University, Via Conca 71, Ancona 60126, Italy; Cardiology and Arrhythmology Clinic, Marche University Hospital, Via Conca 71, Ancona 60126, Italy; Department of Biomedical Sciences and Public Health, Marche Polytechnic University, Via Conca 71, Ancona 60126, Italy; Cardiology and Arrhythmology Clinic, Marche University Hospital, Via Conca 71, Ancona 60126, Italy; Arrhythmia and Electrophysiology Center, IRCCS Policlinico San Donato, San Donato Milanese, Milano, Italy; Vita-Salute San Raffaele University, Cardio-Thoracic-Vascular Departmente, via Olgettina 58, Milano 20132, Italy; Department of Clinical Electrophysiology and Cardiac Pacing, Centro Cardiologico Monzino IRCCS, Milan, Italy; Department of Biomedical Sciences and Public Health, Marche Polytechnic University, Via Conca 71, Ancona 60126, Italy; Cardiology and Arrhythmology Clinic, Marche University Hospital, Via Conca 71, Ancona 60126, Italy; Cardiology Department, Mediterranea Cardiocentro, Via Orazio 2, Naples 80131, Italy; Arrhythmia and Electrophysiology Center, IRCCS Policlinico San Donato, San Donato Milanese, Milano, Italy; Vita-Salute San Raffaele University, Cardio-Thoracic-Vascular Departmente, via Olgettina 58, Milano 20132, Italy; Department of Cardiology, General Regional Hospital “F. Miulli”, Acquaviva delle Fonti, Bari, Italy; De Gasperis Cardio Center, Electrophysiology Unit, Niguarda Hospital, Milan, Italy; Heart Rhythm Management Centre, Postgraduate Program in Cardiac Electrophysiology and Pacing, Universitair Ziekenhuis Brussel - Vrije Universiteit Brussel, Brussels, Belgium; Cardiology Department, Guglielmo da Saliceto Hospital Piacenza, Italy; Department of Clinical Electrophysiology and Cardiac Pacing, Centro Cardiologico Monzino IRCCS, Milan, Italy; Division of Cardiology, Cardiovascular and Thoracic Department, Città della Salute e della Scienza Hospital, Turin, Italy; Department of Medical Sciences, University of Turin, Turin, Italy; Azienda Sanitaria Alto Adige, Cardiology and Functional Tests, Bolzano, Italy; Cardiology Department, Guglielmo da Saliceto Hospital Piacenza, Italy; De Gasperis Cardio Center, Electrophysiology Unit, Niguarda Hospital, Milan, Italy; Cardiology Department, Mediterranea Cardiocentro, Via Orazio 2, Naples 80131, Italy; Department of Clinical, Special, and Dental Sciences, Marche Polytechnic University, Ancona, Italy; Maria Cecilia Hospital, GVM Care & Research, Department of cardiology and cardiac surgery, Cotignola, Italy; Department of Cardiology, General Regional Hospital “F. Miulli”, Acquaviva delle Fonti, Bari, Italy; Department of Medicine, LUM University, Casamassima (BA), Italy; Department of Clinical Electrophysiology and Cardiac Pacing, Centro Cardiologico Monzino IRCCS, Milan, Italy; Department of Biomedical, Surgical and Dental Sciences, University of Milan, Milan, Italy; Arrhythmia and Electrophysiology Center, IRCCS Policlinico San Donato, San Donato Milanese, Milano, Italy; Vita-Salute San Raffaele University, Cardio-Thoracic-Vascular Departmente, via Olgettina 58, Milano 20132, Italy

**Keywords:** Atrial fibrillation, Pulsed field ablation, Catheter ablation, Variable-loop circular catheter

## Introduction

Over the past few years, pulsed field ablation (PFA) has been adopted as a minimally thermal, tissue-selective approach.^1,2^ In 2024, a novel integrated PFA platform (VARIPULSE™) was introduced for atrial fibrillation (AF) ablation. While preliminary multicentre studies have reported encouraging results, real-world data on its safety and efficacy remain scarce. This registry aims to evaluate a focus safety and efficiency of VARIPULSE.

## Methods

### Patients

We conducted an observational, multicentre study to report on the real-world experience with the variable-loop circular catheter (VLCC). Nine Italian arrhythmology centres were asked to collect data on AF ablation procedures. The study complied with the Helsinki Declaration. Patients provided informed consent.

### Ablation

The VARIPULSE platform consists of the VLCC and a multichannel PFA generator (TRUPULSE). The TRUPULSE can release short-duration, high-voltage bipolar biphasic pulses over a few seconds (1800 V in a sequence of three applications, 250 ms each, spaced out by a time interval of 10 s for a total PFA time of ∼21 s) to the VLCC, an open-irrigated bidirectional catheter.^3^

For each pulmonary vein (PV), at least four ablations were performed: two with a closed-loop (25 mm) and two with an open-loop (35 mm). Additional ablations were performed at the operator’s discretion. The ablation procedure was performed as previously described in other studies, using 4 mL/min irrigation settings.^4,5^ Periprocedural anticoagulation was managed by administering an initial bolus of 10 000–12 000 IU of unfractionated heparin, followed by continuous infusion. Prior to ablation delivery, the activated clotting time (ACT) was confirmed to be >350 s.

### Endpoints-statistics

The primary endpoint of the study was to evaluate the safety of the VLCC; complications were classified according to the EHRA/HRS classification.^6^ The secondary endpoint was to assess acute procedural efficiency. Additional secondary endpoints were to evaluate the same parameters across paroxysmal AF vs. persistent AF ablation, and PV isolation (PVI)-only vs. PVI-plus ablation. Statistical analyses were performed using the Statistical Package for the Social Sciences (SPSS®).

## Results

A total of 361 patients were enrolled between April 2024 and July 2025 across nine high-volume tertiary referral electrophysiology centres. All procedures were performed by electrophysiologists with at least 10 years of experience in AF ablation. Clinical and echocardiographic characteristics are summarized in *Table 1*.

**Table 1:** Baseline clinical, echocardiographic and procedural characteristics of enrolled patients.

	VARIPULSE (*N* = 361)		VARIPULSE (*N* = 361)	Paroxysmal AF (*N* = 215)	Persistent AF (*N* = 146)	*P* value	PV only (*N* = 190)	PVI plus (*N* = 171)	*P* value
**Age, median [IQR]**	63 [58–69]	–VARIPULSE, *n* (%)	230 (64)	123 (57)	107 (73)	0.002	113 (60)	117 (68)	0.078
		–Pentaray, *n* (%)	23 (6)	22 (10)	1 (1)	<0.001	9 (5)	14 (8)	0.261
		–**Octaray, *n* (%)**	25 (7)	8 (4)	17 (12)	0.007	13 (7)	12 (7)	0.887
		–**Soundfam, *n* (%)**	83 (23)	62 (29)	21 (14)	0.002	53 (28)	30 (18)	0.017
**Male sex, *n* (%)**	254 (70)	**Mapping points, median [IQR]**	1296 [563–2393]	1100 [490–2027]	1764 [741–3118]	<0.001	1125 [533–2229]	1403 [630–2725]	0.038
**First-line therapy AF ablation, *n* (%)**	56 (16)	–**LCPV, *n* (%)**	69 (19)	43 (20)	26 (18)	0.603	39 (21)	30 (18)	0.558
		–**RCPV, *n* (%)**	12 (3)	11 (5)	1 (1)	0.045	11 (6)	1 (1)	0.014
**CHA_2_DS_2_-VA, median [IQR]**	1 [1–3]	**3D mapping LA volume (mL), mean (SD)**	112.5 (38.4)	100.6 (30.1)	128.6 (42.4)	<0.001	100.9 (31.3)	125.4 (41.4]	<0.001
**BMI, median [IQR]**	26.2 [24–28.6]	**PW ablation, *n* (%)**	120 (33)	9 (4)	111 (76)	<0.001	0 (0)	120 (70)	<0.001
**Tachycardio-myopathy, *n* (%)**	20 (6)	**Extra PV/PW ablation**							
		–**MI, *n* (%)**	6 (2)	1 (0.5)	5 (3)	0.082	0 (0)	6 (4)	0.027
		–**CTI, *n* (%)**	18 (5)	6 (3)	12 (8)	0.038	0 (0)	18 (11)	<0.001
		–**AW, *n* (%)**	114 (32)	23 (11)	91 (62)	<0.001	0 (0)	114 (67)	<0.001
		–**LAA, *n* (%)**	18 (5)	0 (0)	18 (12)	<0.001	0 (0)	18 (11)	<0.001
		–**SVC, *n* (%)**	1 (0.3)	0 (0)	1 (0.7)	0.224	0 (0)	1 (0.6)	0.958
**Paroxysmal AF, *n* (%)** **Early-Persistent AF, *n* (%)** **Persistent AF, *n* (%)** **Long-standing persistent AF, *n* (%)**	215 (59) 30 (8) 67 (19) 49 (14)	**PFA app.:**							
		**−LSPV, median [IQR]**	15 [12–18]	15 [12–18]	12 [12–15]	<0.001	15 [12–18]	12 [12–15]	0.005
		**−LIPV, median [IQR]**	12 [12–15]	12 [12–15]	12 [9–15]	0.003	12 [12–15]	12 [9–15]	0.006
		**−LCPV, median [IQR]**	26 [21–28]	27 [24–30]	21 [18–27]	0.042	27 [24–30]	24 [18–27]	0.168
		**−RSPV, median [IQR]**	14 [12–15]	15 [12–15]	12 [12–15]	0.013	14.5 [12–15]	12 [12–15]	0.156
		**−RIPV, median [IQR]**	12 [12–15]	13 [12–15]	12 [12–15]	0.004	14 [12–15]	12 [12–15]	<0.001
		**−PW, median [IQR]**	24 [18–30]	21 [9–26]	24 [18–32]	0.190		24 [18–30]	
		**−Extra PV/PW, median [IQR]**	18 [12–30]	9 [6–33]	18 [15–30]	0.062		18 [12–30]	
**Mild MR, *n* (%)** **Moderate-severe MR, *n* (%)**	167 (47) 45 (13)	**Tot. no. of app., median [IQR]**	63 [54–84]	57 [54–69]	84 [66–105]	<0.001	56.5 [51–63]	85 [72–105]	<0.001
**Hypertension, *n* (%)**	209 (58)	**Complete PVI, *n* (%)**	352 (98)	212 (99)	140 (96)	0.105	185 (97)	167 (97)	0.578
**Diabetes, *n* (%)**	32 (9)	**Mapping (min), median [IQR]**	6 [5–11]	6 [5–10]	7 [5–12]	0.011	6 [5–10]	8 [5–13]	<0.001
**Smoke, *n* (%)**	47 (13)	**LA dwell time (min), median [IQR]**	35 [29–45]	35 [25–43]	39 [33–48]	<0.001	31 [25–40]	40 [35–50]	<0.001
**Vascular disease/CAD, *n* (%)**	37 (10)	**Skin-to-skin time (min), median [IQR]**	61.5 [54–75]	61 [54–75]	62 [54–77.8]	0.497	60 [50–70]	66 [55–80]	0.001
**OSAS, *n* (%)**	26 (7)	**Fluoro time (min), median [IQR]**	7 [5–12]	8 [5–13]	7 [4–11]	0.441	8 [5–13]	7 [4–11]	0.263
**Prior stroke/TIA, *n* (%)**	13 (4)	**Recurrence during hospitalization, *n* (%)**	16 (4)	4 (2)	12 (8)	0.009	3 (2)	13 (8)	0.012
**LVEF %, median [IQR]**	60 [55–61]	**Minor complications, *n* (%)**	7 (2)	4 (2)	3 (2)	0.847	3 (2)	4 (2)	0.611
**iLAV (mL/m^2^), mean (SD)**	36.5 (10.5)	**Major complications, *n* (%)**	2 (0.6)	0 (0)	2 (1)	0.224	0 (0)	2 (1)	0.291

The table summarizes the baseline clinical, echocardiographic, and procedural characteristics of the patients included in the study. Abbreviations: AF, atrial fibrillation; App., applications; AW, anterior wall; BMI, body mass index; CAD, coronary artery disease; CTI, cavo-tricuspid isthmus; fluoro, fluoroscopy; LA, left atrial; LAA, left atrial appendage; iLAV, indexed left atrial volume; IQR, interquartile range; LIPV, left inferior pulmonary vein; LSPV, left superior pulmonary vein; LVEF, left ventricular ejection fraction; MI, mitral isthmus; MR, mitral regurgitation; *N*, number; OSAS, obstructive sleep apnoea syndrome; PFA, pulsed field ablation; PV, pulmonary vein; PVI, pulmonary vein isolation; PW, posterior wall; RIPV, right inferior pulmonary vein; RSPV, right superior pulmonary vein; SVC, superior vena cava; TIA, transient ischaemic attack.

### Complication

A total of nine procedure-related complications were observed, corresponding to an overall 2.5% rate. Two major complications [0.2%; one post-procedural ischaemic stroke and one transient ischaemic attack (TIA)] and seven (2%) minor complications occurred.

The ischaemic stroke occurred in a patient with persistent AF who underwent an extensive ablation strategy, including isolation of both the anterior and posterior wall in addition to PVI (78 applications). The patient had been maintained on uninterrupted, appropriately dosed oral anticoagulation, and pre-procedural transoesophageal echocardiography had excluded left atrial (LA) thrombus. Critically, even though all participating centres followed the same protocol described above, PFA applications were delivered without confirming an ACT >350 s.

The TIA occurred in a patient with paroxysmal AF treated using a PVI-only approach; brain magnetic resonance imaging and CT scan showed no structural abnormalities; no specific intervention beyond diagnostic evaluation was required.

Minor complications included six vascular access–related events (two deep venous thrombosis, three small arteriovenous fistulas, and one pseudoaneurysm; all vascular accesses were obtained under ultrasound guidance by electrophysiology fellows/trainees), none of which required surgical intervention, and one transient episode of coronary spasm, which occurred during PFA delivery in the posterior wall and resolved after nitroglycerine infusion.

### Procedural data

Procedural data were analysed for the overall cohort and across four predefined subgroups: patients with PAF vs. persistent AF and patients treated with a PVI-only approach vs. those receiving ablation at extra-PV sites. Procedures in persistent AF patients involved a more extensive lesion set and a greater number of applications and were associated with longer mapping/LA-dwell times (*Table 1*).

## Discussion

### Safety

Overall complication rates were low despite the inclusion of complex lesion sets, consistent with the inspIRE/admIRE trials and recent observational registries.^4–10^

On 17 February 2025, a field safety notice from Biosense Webster reported an increased stroke risk in persistent AF, particularly with a high number of applications, when overlapping and extending beyond the PV. The VARIPULSE system is CE-marked for atrial fibrillation, and there are no specific contraindications for extended extra-PV ablation. Regarding the maximum number of applications, the lowest number of lesions is generally recommended. Both 16 and 23 deliveries (48 and 69 applications) have been shown to be safe; beyond this threshold, a 50% incidence of cerebral ischaemic events was reported.^8^

The stroke occurred in a procedural context characterized by subtherapeutic intraprocedural anticoagulation and a high number of extra-PV applications in the setting of persistent AF. This may therefore be consistent with the scenario described in the safety notice, although several other cases in our series involved a substantially higher lesion burden.^1–4^ Importantly, these procedures were performed with a low irrigation flow rate of 4 mL/min. More recent evidence suggests that increasing irrigation to 30 mL/min may further reduce thrombus formation,^7,10^ and future refinements in PFA waveform design may further improve safety. Recent real-world data, including the European VARIPURE registry (0.6% primary adverse event rate, no strokes)^3^ and the REAL-AF registry analysis of VLCC (acute complication rate 0.4%), support a highly favourable safety profile.^5^

### Efficiency

Procedural parameters were excellent across paroxysmal AF and persistent AF cases and in both PVI-only and additional-lesion procedures. Fluoroscopy time, LA dwell time, and total skin-to-skin procedural duration were within the ranges reported for contemporary PFA systems.^2–5,7–10^

The VLCC demonstrated high efficiency and versatility for performing additional extra-PV lesions. The availability of the integrated electroanatomical mapping system enabled acute validation of lesions.^3,4^

Some limitations should be acknowledged, primarily the absence of long-term follow-up, which prevents a formal assessment of clinical efficacy in terms of arrhythmia recurrence. Another limitation is that all procedures were performed at tertiary referral centres by experienced operators, potentially biasing the observed safety profile.

## Conclusion

VLCC demonstrated a favourable safety profile and high procedural efficiency across a wide spectrum of AF ablation scenarios. The low observed complication rate suggests an overall favourable safety profile. However, it should be noted that an ischaemic stroke occurred in a procedure in which the proposed protocol was not fully adhered to.
